# Multimodal data fusion of dual-modal DWI-ADC MRI and clinical variables for prognostic prediction in acute ischemic stroke

**DOI:** 10.3389/fneur.2026.1849525

**Published:** 2026-06-16

**Authors:** Yanliang Ji, Xuewen Wo, Bo Yuan, Yaran Liu, Zhidong Xue

**Affiliations:** 1 Shandong Key Lab of Complex Medical Intelligence and Aging, Shandong Medical and Pharmaceutical University, Yantai, China; 2 The Second Clinical Medical College of Shandong Medical and Pharmaceutical University, Yantai, China; 3 Department of Neurology, Binzhou People’s Hospital Affiliated to Shandong First Medical University, Binzhou, China; 4 Institute of Artificial Intelligence, Beihang University, Beijing, China; 5 Beijing Advanced Innovation Center for Future Blockchain and Privacy Computing, Beihang University, Beijing, China; 6 School of Health Management, Shandong Medical and Pharmaceutical University, Yantai, China; 7 Huazhong University of Science and Technology, Wuhan, China

**Keywords:** acute ischemic stroke, clinical risk modeling, dual-modal magnetic resonance imaging, multimodal fusion, prognostic prediction

## Abstract

**Objectives:**

To develop and internally evaluate a probability-level stacked ensemble model integrating diffusion-weighted imaging (DWI) and apparent diffusion coefficient (ADC) magnetic resonance imaging (MRI)-based imaging predictions with routinely available clinical predictions to predict 90-day poor functional outcome in patients with acute ischemic stroke.

**Methods:**

This retrospective single-center study included 562 patients with acute ischemic stroke who underwent DWI and ADC MRI within 1 to 7 days after symptom onset. Poor outcome was defined as 90-day modified Rankin Scale >2. Patients were assigned at the patient level to training (*n* = 393), validation (*n* = 84), and held-out internal test (*n* = 85) sets. A ResNet50-based imaging model and a support vector regression (SVR)-based clinical model were integrated using logistic regression as a probability-level meta-learner. Model performance was evaluated using the area under the receiver operating characteristic curve (AUC), sensitivity, specificity, calibration, decision curve analysis, and the DeLong test.

**Results:**

In the held-out internal test set, the fusion model achieved the highest numerical AUC (0.951, 95% CI: 0.908–0.994), with sensitivity of 0.889 and specificity of 0.911. The DeLong test showed that the fusion model significantly outperformed the imaging model (*p* < 0.05), but not the clinical model or Wouters 2018 model. Brier scores were 0.077 for the Wouters 2018 model, 0.089 for the clinical model, 0.094 for the fusion model, and 0.122 for the imaging model. Decision curve analysis showed that the fusion model had positive net benefit across threshold probabilities from 0.10 to 0.60 and exceeded the imaging model, but did not consistently exceed the clinical model or Wouters 2018 model.

**Conclusion:**

The fusion model showed high internal discrimination and improved performance compared with imaging alone, but its incremental value over clinical models was limited in calibration and decision curve analyses. External validation, recalibration, and prospective evaluation are required before broader clinical use.

## 1 Introduction

Acute ischemic stroke (AIS) has an abrupt onset (1, 2). It frequently results in irreversible neurological damage, imposing a substantial burden on patients, their families, and healthcare systems (3). Early prediction of functional outcomes may support risk stratification, rehabilitation planning, and healthcare resource allocation, particularly when the prediction target is clearly defined and model uncertainty is appropriately considered (4). Magnetic resonance imaging (MRI), with its multi-sequence capabilities, provides robust technical support for the early assessment of AIS. Among these sequences, diffusion-weighted imaging (DWI) enables the precise delineation of the distribution of acute ischemic lesions, while the apparent diffusion coefficient (ADC) map quantitatively characterizes the degree of restricted diffusion of water molecules in brain tissue (4, 5). The combined use of DWI and ADC can provide complementary information on lesion distribution and tissue diffusion restriction, which may be relevant for functional outcome prediction (6). Previous studies have explored clinical models, radiomics models, and deep learning models for AIS outcome prediction, including approaches that combine imaging and clinical information (7). Nevertheless, some existing approaches still depend on manually defined imaging descriptors, selected imaging sequences, or fusion strategies that do not explicitly separate imaging and clinical contributions. Therefore, further evaluation of DWI-ADC MRI features together with routinely available clinical variables remains clinically and methodologically relevant (8).

The 90-day modified Rankin Scale (mRS) score is currently the internationally recognized gold standard for assessing long-term functional outcomes in patients with AIS (9, 10). Several clinical prediction models, including the model developed by Zhang et al. (9), have shown that routinely available clinical variables such as age and National Institutes of Health Stroke Scale (NIHSS) scores are informative for 90-day mRS prediction. However, clinical variables alone may not fully reflect lesion location, lesion extent, and tissue-level imaging heterogeneity (11). Recent studies have increasingly incorporated imaging and clinical information into AIS prognostic models. For example, Xie et al. (2) combined imaging, demographic, and clinical information using machine learning, and Brugnara et al. (12) developed multimodal predictive models for functional outcomes after endovascular treatment. More recent studies have also explored multimodal ensemble deep learning, radiomics, and deep learning frameworks for predicting functional outcomes or poor prognosis after AIS (13, 14). These studies support the value of multimodal modeling, while also indicating that modality selection, fusion strategy, validation design, and interpretability remain important issues for model development (15).

Convolutional neural networks can reduce reliance on manual feature engineering by learning task-specific imaging representations from MRI data (16). In the present study, probability-level stacking was used to integrate imaging and clinical predictions rather than directly concatenating high-dimensional features (17). This strategy was selected because DWI-ADC images and clinical variables differ substantially in data structure, dimensionality, and statistical distribution. A probability-level design also allows the imaging and clinical branches to be trained and evaluated separately, thereby improving transparency and reducing model complexity in a moderate-sized single-center cohort (18). ResNet50 was selected as the imaging backbone because it provides a well-established convolutional architecture suitable for transfer learning with limited medical imaging data (19). The support vector regression (SVR)-based clinical branch was used to generate a continuous clinical risk score from routinely available variables, and logistic regression was used as the meta-learner because it provides a simple and interpretable probability-level fusion mechanism.

Accordingly, this retrospective single-center study developed and internally evaluated a stacked ensemble model integrating DWI-ADC MRI features and routinely available clinical variables for predicting 90-day poor functional outcome in patients with AIS. The intended use of the model is early risk stratification after baseline clinical assessment and MRI acquisition, rather than direct treatment decision-making. The main objectives were to evaluate the performance of a probability-level fusion strategy, compare it with imaging-only, clinical, and published clinical baseline models, and examine the contribution of clinical variables using SHapley Additive ExPlanations (SHAP) analysis.

## 2 Materials and methods

### 2.1 Study population and MRI data acquisition

**Figure 1:**
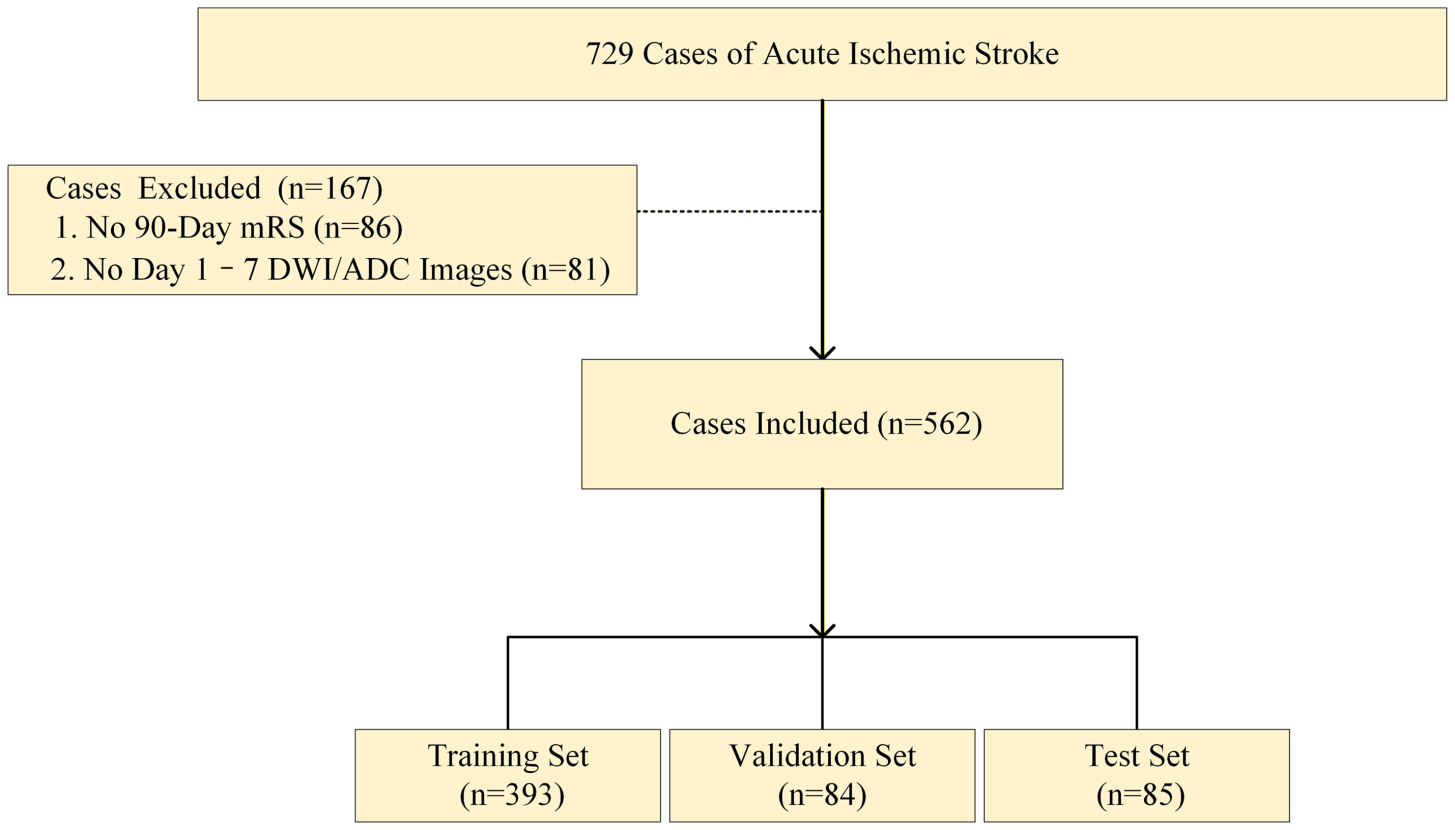
Flowchart of patient selection and dataset partitioning process.

This retrospective study was conducted in accordance with the Declaration of Helsinki and was approved by the Ethics Committee of Binzhou People’s Hospital Affiliated to Shandong First Medical University (Approval No.: YXKYLL-20251108). Patients with AIS were diagnosed according to established criteria (20). Consecutive patients with AIS admitted between January 2024 and May 2025 were screened for eligibility. The inclusion criteria were as follows: (1) MRI examination including DWI and ADC sequences performed within 1–7 days after symptom onset and (2) the availability of complete 90-day mRS follow-up data. A total of 729 patients with AIS were initially screened. Among them, 86 were excluded due to missing 90-day mRS scores, and 81 were excluded because DWI or ADC images within 1–7 days after onset were unavailable. Finally, 562 patients were included and randomly assigned at the patient level to the training set (*n* = 393), validation set (*n* = 84), or held-out internal test set (*n* = 85) at a ratio of 7:1.5:1.5. The split was stratified according to the 90-day mRS outcome to maintain a comparable proportion of poor outcomes across the three subsets. All DWI and ADC images from the same patient were assigned to the same subset, and no image from one patient was allowed to appear in more than one subset. The 7:1.5:1.5 ratio was selected to retain sufficient samples for model fitting while preserving separate validation and internal testing sets. The validation set was used for hyperparameter selection, learning rate scheduling, early stopping, and meta-learner training, whereas the held-out internal test set was used only for final model evaluation. Figure 1 shows a flowchart of patient selection and the data partitioning process.

### 2.2 MRI data preprocessing

All preprocessing procedures for DWI and ADC images were implemented using PyTorch and torchvision. All images were loaded using PIL and converted to RGB format. For grayscale MRI images, this conversion replicated the single intensity channel into three channels to match the input requirement of the ImageNet-pretrained ResNet50 backbone. Images were resized to 224 × 224 pixels, converted to tensors, and normalized using ImageNet mean and standard deviation values. This normalization was used to maintain compatibility with the pretrained network weights rather than to assume that MRI intensities follow the distribution of natural images.

Data augmentation was applied only after patient-level data partitioning and only to images in the training set. No augmentation was performed for the validation set or the held-out internal test set. This procedure ensured that augmented images derived from one patient could not enter different data subsets and that model evaluation was performed using unaugmented clinical MRI inputs. During imaging model training, individual DWI and ADC images were used as image-level inputs, while the outcome label was inherited from the corresponding patient. During inference, predictions were aggregated at the patient level. Specifically, logits from all eligible images of the same patient were averaged and then converted into a patient-level probability using the sigmoid function. The patient-level probability was used for receiver operating characteristic (ROC) analysis, calibration analysis, decision curve analysis, and threshold-based classification.

### 2.3 Clinical variables and outcome definition

Clinical variables were extracted from electronic medical records, including baseline NIHSS score, serum D-dimer level, sex, medical history, aphasia, and cognitive impairment. Medical history was defined as a composite variable reflecting the presence of previous stroke, hypertension, or diabetes mellitus. These variables were prespecified before model training based on clinical relevance, routine availability, and evidence from previous stroke prognostic studies. No validation set or held-out internal test set information was used for feature selection. After patient-level data splitting, missing values in clinical predictors were handled within the preprocessing pipeline. Continuous variables were imputed using the mean value estimated from the training set and then standardized using training-set parameters. Categorical variables were imputed using the most frequent category estimated from the training set and then encoded using one-hot encoding. The fitted preprocessing pipeline was applied unchanged to the validation and held-out internal test sets. No outcome information from the validation or held-out internal test set was used for imputation, feature selection, or model tuning.

The primary outcome was functional status 90 days after stroke onset, assessed using mRS scores. A poor outcome was defined as an mRS score >2 (21), indicating moderate-to-severe disability or death, whereas a favorable outcome was defined as an mRS score ≤2, indicating functional independence, consistent with established conventions (22, 23). Complete 90-day mRS follow-up data were available for all patients and were used as the reference standard for model development and evaluation (24).

### 2.4 Model architecture and training strategy

**Figure 2:**
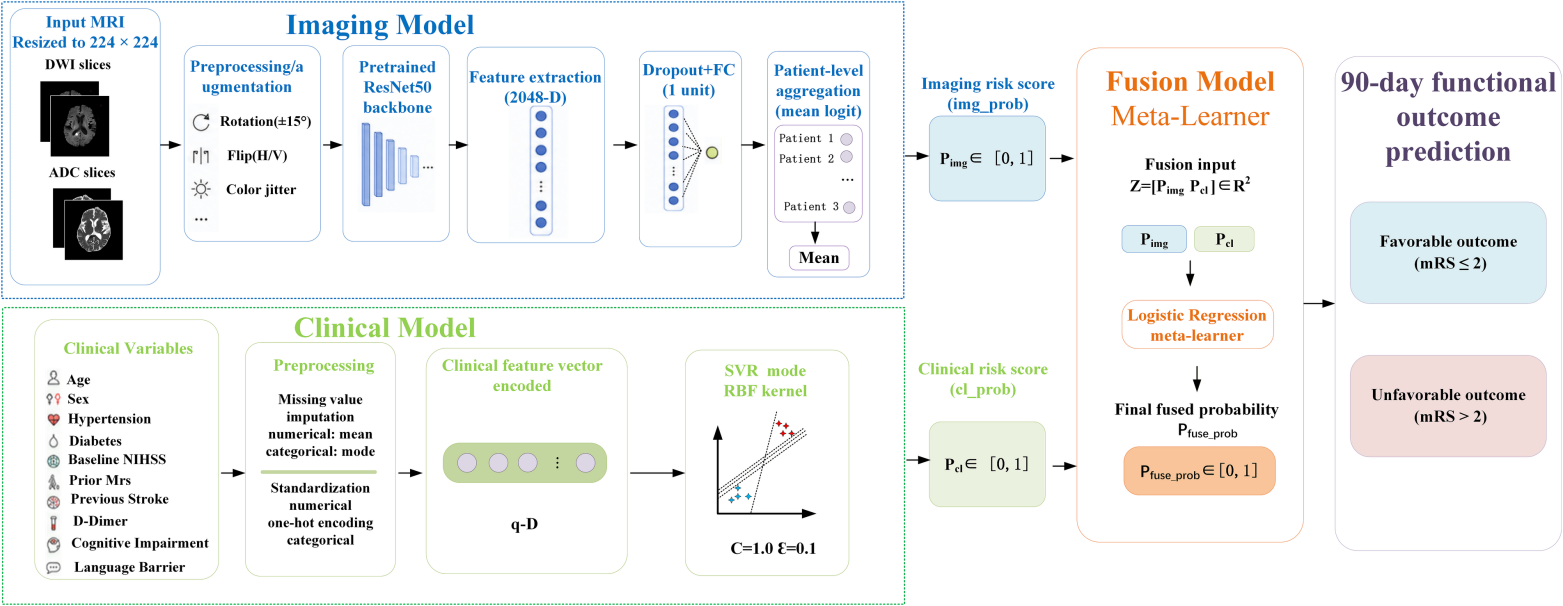
Framework of the probability-level stacked fusion model integrating DWI-ADC MRI-based imaging predictions and routinely available clinical predictions.

A probability-level stacked ensemble model was developed to integrate imaging predictions derived from DWI and ADC MRI and clinical predictions derived from routinely available variables for 90-day outcome risk stratification in patients with AIS. The model consisted of two independently trained base learners, namely a ResNet50-based imaging branch and an SVR-based clinical branch, followed by a logistic regression meta-learner. The predicted probabilities of poor outcome generated by these base learners were used as intermediate features and subsequently fused at the probability level using a logistic regression meta-learner, with a dichotomization threshold of 0.5 for the final prediction. The overall framework is illustrated in Figure 2.

Probability-level stacking was selected because the imaging and clinical branches generated heterogeneous predictors with different feature spaces and scales. Compared with direct feature concatenation, this design allowed each modality-specific branch to be optimized and evaluated separately. Compared with more parameter-intensive attention-based fusion modules, the logistic regression meta-learner provided a simpler fusion strategy that was considered more suitable for the moderate size of the present single-center cohort.

The clinical branch was constructed using support vector regression with a radial basis function kernel to generate a continuous clinical risk score for poor 90-day outcome. Although the final task was binary classification, SVR was used to model continuous risk tendency. The resulting scores were clipped to the [0, 1] range and treated as probability-like risk estimates for ROC analysis, calibration assessment, and model fusion. The preprocessing pipeline included mean imputation and standardization for continuous variables and most frequent category imputation and one-hot encoding for categorical variables. Hyperparameters were selected using the training and validation sets without accessing the held-out internal test set. The final model used C = 1.0, epsilon = 0.1, and gamma = scale. The model was implemented using Scikit-learn. Following model training, SHAP values were used to assess the contribution of each clinical variable to the predicted risk of poor outcome.

For DWI and ADC images, a ResNet50 architecture pretrained on ImageNet was used as the imaging backbone. To adapt the pretrained model to the AIS prognosis task while limiting overfitting, all backbone parameters except those in the final 10 layers were frozen, and only the final 10 layers and the newly added classification head were updated during training. The original fully connected layer was replaced with a dropout layer and a one-dimensional fully connected layer to generate binary logits. The imaging branch was optimized using AdamW with an initial learning rate of 1 × 10^−4^ and weight decay of 1 × 10^−5^. A ReduceLROnPlateau scheduler monitored validation area under the receiver operating characteristic curve (AUC). The dropout rate in the classification head was 0.5. Binary cross-entropy loss with logits was used. No oversampling, undersampling, or synthetic sample generation was applied to the training, validation, or held-out internal test sets. No class weighting or balanced sampling strategy was used during model training. To address the imbalanced outcome distribution at the evaluation stage, sensitivity, specificity, calibration metrics, decision curve analysis, and confusion matrices were reported in addition to AUC. The batch size was 16, and the maximum number of epochs was 50. The model checkpoint with the highest validation AUC was selected for final evaluation on the held-out internal test set. Mixed precision training was used to improve computational efficiency.

During inference, logits from all eligible images of each patient were averaged first and then converted into a patient-level probability using the sigmoid function. A threshold of 0.5 was used for the primary binary classification analysis, while threshold-independent evaluation was performed using ROC analysis.

Logistic regression was employed as the meta-learner and trained on the predicted probabilities generated by the clinical and imaging branches to perform probability-level fusion. To avoid information leakage at the stacking stage, the meta-learner was fitted using validation set predictions from the two base learners. The held-out internal test set was used only for generating final fused probabilities and evaluating model performance. The logistic regression model was implemented using Scikit-learn with L2 regularization, the liblinear solver, a maximum of 1,000 iterations, and a fixed random seed of 42 to ensure reproducibility. The final fused probabilities were constrained to the [0, 1] range using np.clip and dichotomized at a threshold of 0.5 for binary classification.

The Wouters 2018 model was selected as the baseline comparator (25). This model was developed by Wouters et al. (25) using multivariable logistic regression, with the change in NIHSS score from baseline to 24 h after onset as the core feature for predicting the functional outcome 90 days after onset.

### 2.5 Performance evaluation

Model performance was evaluated in terms of discrimination, threshold-based classification, calibration, and clinical utility. Discrimination was assessed using the area under the receiver operating characteristic curve. Threshold-based classification performance was evaluated using sensitivity, specificity, and confusion matrices at the predefined threshold of 0.5. Confusion matrices were generated for the Wouters 2018 model, clinical branch, imaging branch, and fusion model in the held-out internal test set. The 95% confidence intervals (CIs) for AUC, sensitivity, specificity, and other classification metrics were estimated using patient-level bootstrap resampling with 1,000 iterations. Calibration was assessed using the Brier score and calibration plots comparing predicted and observed risks. Decision curve analysis was performed to evaluate the net benefit of each model across clinically relevant threshold probabilities (26). The Wouters 2018 model, clinical branch, imaging branch, and fusion model were evaluated using the same patient-level outcome labels and the same held-out internal test set.

### 2.6 Model interpretability analysis

Model interpretability was assessed separately for the clinical and imaging branches. For the clinical branch, SHAP values were calculated to estimate the contribution of each clinical variable to the predicted risk of poor outcome. This analysis was intended to interpret the clinical branch rather than the complete fusion framework. The visualization focused on two aspects: the average magnitude of each feature’s impact on model predictions and the relationship between feature values and the direction of their contributions.

For the imaging branch, channel-wise averaged activation heatmaps were generated from intermediate convolutional layers to provide qualitative visualization of model response patterns. Activation heatmaps were generated for multiple representative cases selected from true positive, true negative, false positive, and false negative predictions. These heatmap analyses were considered exploratory and were not interpreted as definitive localization or causal explanations of model decisions.

### 2.7 Statistical analysis

Between-model differences in AUC values were assessed using the DeLong test. To further evaluate the stability of the fixed training-validation-test split, stratified five-fold cross-validation was performed as a supplementary internal robustness analysis. Model development procedures, including preprocessing, base learner training, meta-learner fitting, and performance evaluation, were repeated within each fold. The held-out internal test set results were retained as the primary performance analysis, and the five-fold cross-validation results were reported as supplementary internal validation.

Normality of the continuous variables was evaluated using the Shapiro–Wilk test to guide subsequent analyses. One-way analysis of variance was employed to compare differences among the training, validation, and held-out internal test sets. For comparisons between patients with favorable and poor outcomes, the Welch *t*-test was applied to normally distributed variables, whereas the Mann–Whitney U test was used for non-normally distributed variables. Categorical variables are presented as counts and percentages (*n* [%]), with group comparisons performed using the chi-square test.

Subgroup analysis was conducted according to sex, age group, baseline NIHSS score group, medical history, and aphasia. To assess potential subgroup-specific variation in model performance, formal interaction testing was performed using logistic regression models with poor 90-day outcome as the dependent variable and model-predicted risk, subgroup variable, and their interaction term as independent variables. For binary subgroup variables, the *p* value for the interaction term was reported. For subgroup variables with more than two categories, an overall interaction test was performed using a likelihood ratio test comparing models with and without the interaction terms. Because the NIHSS >15 subgroup contained a limited number of patients and a very high event rate, baseline NIHSS severity was dichotomized as 0 to 4 versus >4 in the formal interaction test, while three-level NIHSS categories were retained for descriptive clinical concordance analysis. This analysis was considered exploratory because some subgroups contained limited numbers of poor outcome events.

In addition to interaction testing, subgroup-based clinical concordance was assessed by comparing the observed incidence of poor outcome with the mean predicted probability generated by the fusion model within each subgroup. This analysis was used to evaluate whether the model generated clinically plausible risk patterns across age, baseline NIHSS score, medical history, aphasia, and sex subgroups. All statistical tests were two-sided, with *p* < 0.05 indicating statistical significance.

## 3 Results

### 3.1 Dataset characteristics and baseline analysis

The patient selection process and data partitioning strategy are presented in the flowchart in Figure 1. A total of 562 patients with AIS were included and assigned at the patient level to the training set (*n* = 393), validation set (*n* = 84), or held-out internal test set (*n* = 85) at a ratio of 7:1.5:1.5. The baseline characteristics of the three cohorts are summarized in Table 1. No significant differences were observed among the three groups in demographic or clinical variables, including age, baseline NIHSS scores, D-dimer levels, sex, medical history, aphasia, and cognitive impairment (*p* > 0.05). These results indicate that the observed baseline characteristics were generally comparable across the three subsets, supporting the use of this split for internal model development and evaluation.

**Table 1:** Comparison of baseline characteristics among training, validation, and held-out internal test sets.

Characteristics	Training set	Validation set	Held-out internal test set	*p*
Age (years)	65.3 ± 11.4	66.4 ± 11.2	67.8 ± 10.0	0.146
NIHSS score	2.6 ± 2.8	2.6 ± 2.5	2.9 ± 3.1	0.577
D-dimer (mg/L)	0.5 ± 0.8	0.4 ± 0.4	0.5 ± 0.6	0.519
Sex (male), *n* (%)	263 (66.9)	54 (64.3)	51 (60.0)	0.462
History of cerebral infarction, *n* (%)	137 (34.9)	29 (34.5)	35 (41.2)	0.527
History of hypertension, *n* (%)	83 (21.1)	18 (21.4)	16 (18.8)	0.884
History of diabetes mellitus, *n* (%)	22 (5.6)	3 (3.6)	3 (3.5)	0.592
Aphasia, *n* (%)	177 (45.0)	39 (46.4)	43 (50.6)	0.647
Cognitive impairment, *n* (%)	5 (1.3)	3 (3.6)	4 (4.7)	0.086
90-day mRS score >2, *n* (%)	80 (20.4)	17 (20.2)	18 (21.2)	0.984

Stratification based on 90-day functional outcome (Table 2) revealed significant differences between the favorable and poor-outcome groups. Patients with poor outcomes exhibited significantly higher baseline NIHSS scores, elevated D-dimer levels, a higher prevalence of aphasia, and a greater proportion of medical history (all *p* < 0.05). In contrast, no significant differences were observed in age, sex, or cognitive impairment between the two groups. These findings are consistent with established clinical evidence and further support the prognostic relevance of the selected variables.

**Table 2:** Comparison of baseline characteristics between patients with favorable and poor prognoses.

Characteristics	90-day mRS score ≤2	90-day mRS score >2	*p*
Age (years)	66.0 ± 10.9	64.3 ± 10.8	0.118
NIHSS score	1.8 ± 1.8	6.1 ± 3.5	<0.001
D-dimer (mg/L)	0.5 ± 0.6	0.7 ± 1.0	<0.001
Sex, *n* (%)			0.538
Female	151 (33.8)	43 (37.4)	
Male	296 (66.2)	72 (62.6)	
Medical history, *n* (%)			<0.001
None	343 (76.7)	18 (15.7)	
With history	104 (23.3)	97 (84.3)	
Aphasia, *n* (%)			<0.001
Yes	167 (37.4)	92 (80.0)	
No	280 (62.6)	23 (20.0)	
Cognitive impairment, *n* (%)			0.489
Yes	11 (2.5)	1 (0.9)	
No	436 (97.5)	114 (99.1)	

### 3.2 Performance of prognostic predictive models

**Figure 3:**
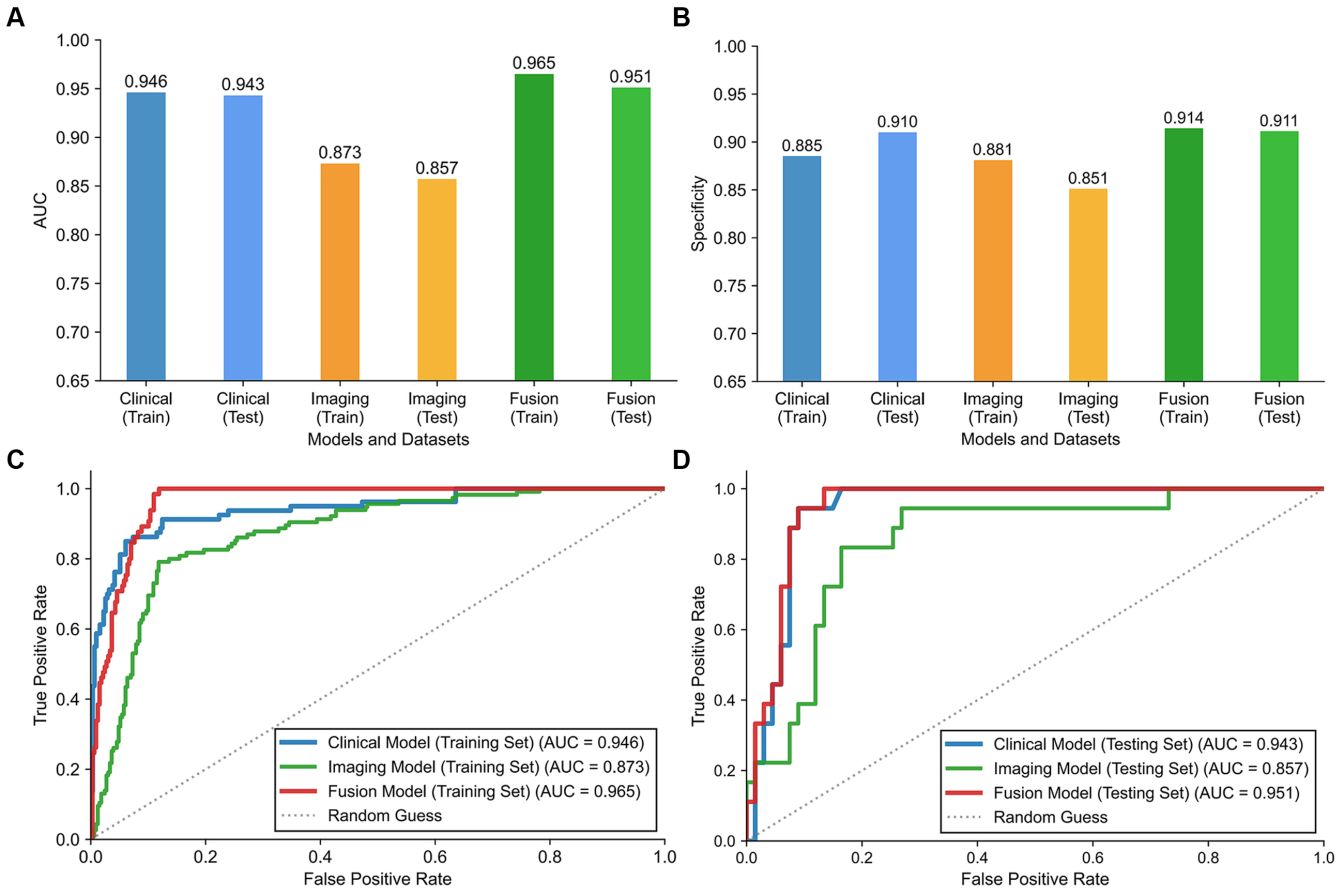
Comprehensive performance evaluation of the prognostic predictive models. **(A)** Bar plot comparing AUC values across models in training and held-out internal test sets. **(B)** Bar plot comparing specificity across models in training and held-out internal test sets. **(C)** ROC curves of the clinical, imaging, and fusion models in the training set. **(D)** ROC curves of the clinical, imaging, and fusion models in the held-out internal test set.

**Figure 4:**
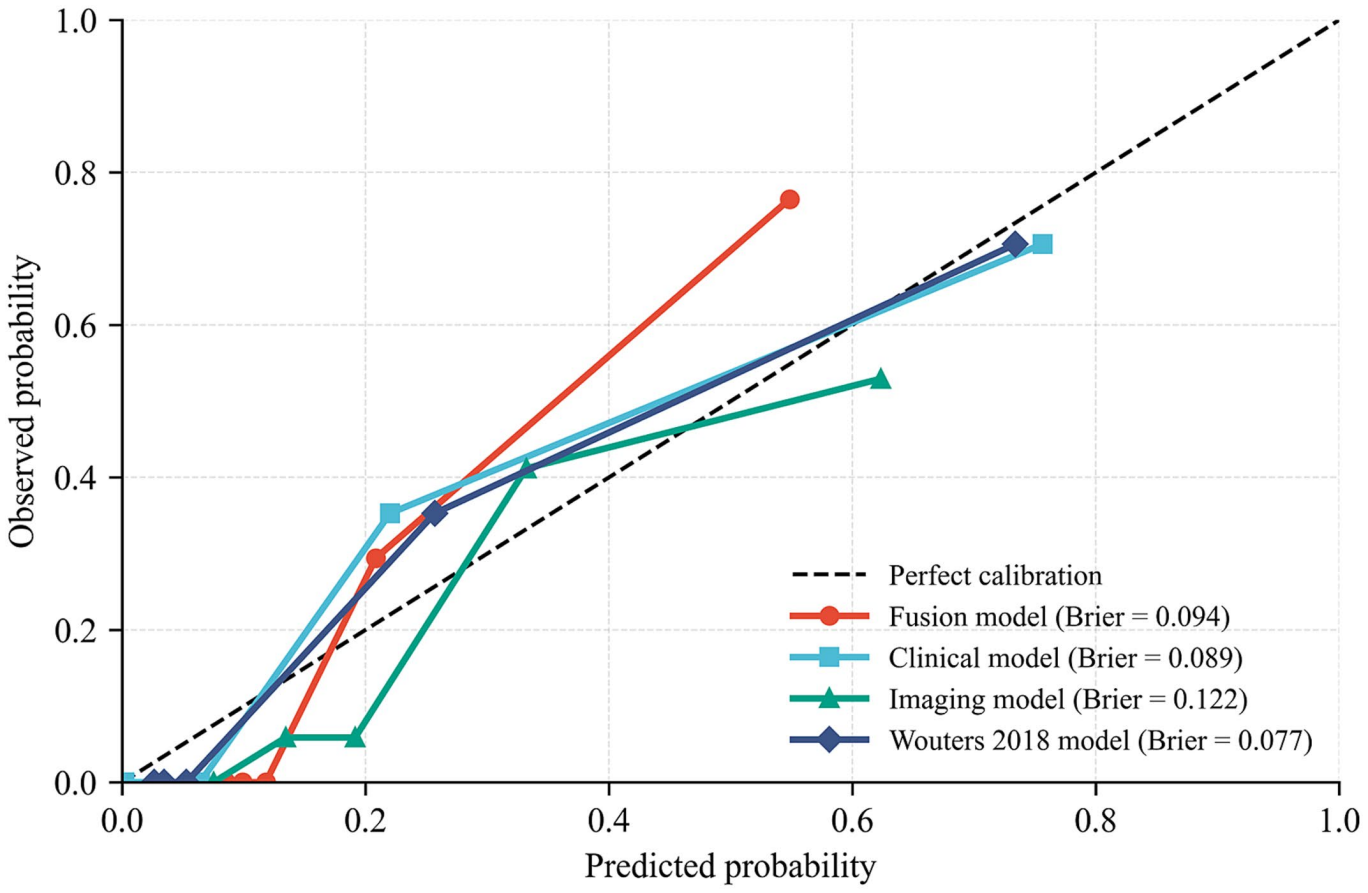
Calibration plots of the four prediction models in the held-out internal test set.

**Figure 5:**
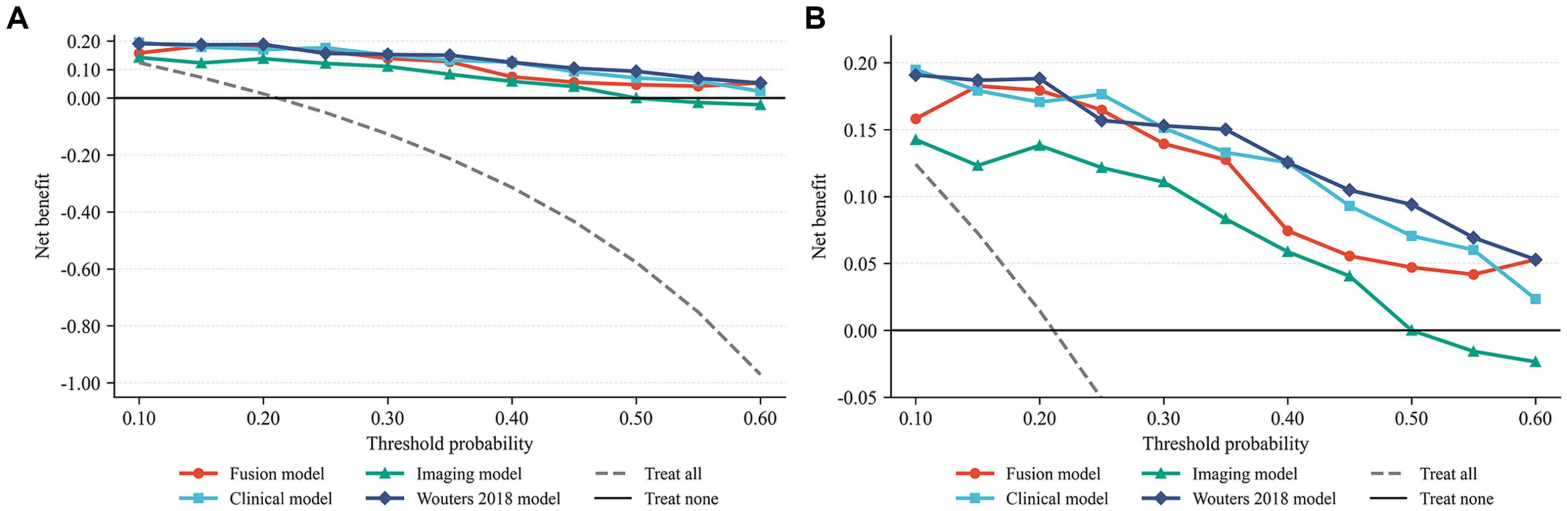
Decision curve analysis of the four prediction models in the held-out internal test set. **(A)** Overview of decision curves. **(B)** Magnified view of panel A highlighting differences among models.

Model performance was evaluated across four domains: discrimination, threshold-based classification, calibration, and clinical utility. Discrimination was assessed using AUC and ROC curves, threshold-based performance was summarized using sensitivity, specificity, and confusion matrices, calibration was assessed using Brier score and calibration plots, and clinical utility was evaluated using decision curve analysis. Figure 3 summarizes the discrimination performance of the clinical model, imaging model, and fusion model, including AUC and specificity comparisons in the training and held-out internal test sets, as well as ROC curves for the two datasets. Calibration and decision curve analyses are presented separately in Figures 4, 5.

#### 3.2.1 Base model performance

The clinical model showed similar discrimination between the training and held-out internal test sets. The AUC was 0.946 (95% CI: 0.912–0.979) in the training set and 0.943 (95% CI: 0.888–0.985) in the held-out internal test set, with a difference of 0.003 (Figure 3A). The specificity was 0.885 in the training set and 0.910 in the held-out internal test set (Figure 3B), whereas the sensitivity was 0.937 and 0.944, respectively. These findings suggest that the clinical model had consistent internal performance when using the selected clinical variables. The imaging model showed lower discrimination than the clinical and fusion models. The AUC was 0.873 (95% CI, 0.833–0.906) in the training set (Figure 3C) and 0.857 (95% CI, 0.764–0.949) in the held-out internal test set (Figure 3A), with corresponding specificities of 0.881 and 0.851 (Figure 3B). The sensitivities were 0.791 and 0.722 in the training and held-out internal test sets, respectively.

#### 3.2.2 Fusion model performance

The fusion model showed high discrimination in the internal evaluation. In the training set, the model achieved an AUC of 0.965 (95% CI: 0.928–0.990) (Figure 3A), with a sensitivity of 0.890 and a specificity of 0.914 (Figure 3B). In the held-out internal test set, the AUC was 0.951 (95% CI: 0.908–0.994), with a sensitivity of 0.889 and a specificity of 0.911. The ROC curves indicate that the fusion model showed numerically higher discrimination than the imaging model and similar discrimination to the clinical model in the held-out internal test set (Figure 3D).

#### 3.2.3 Model comparison

**Figure 6:**
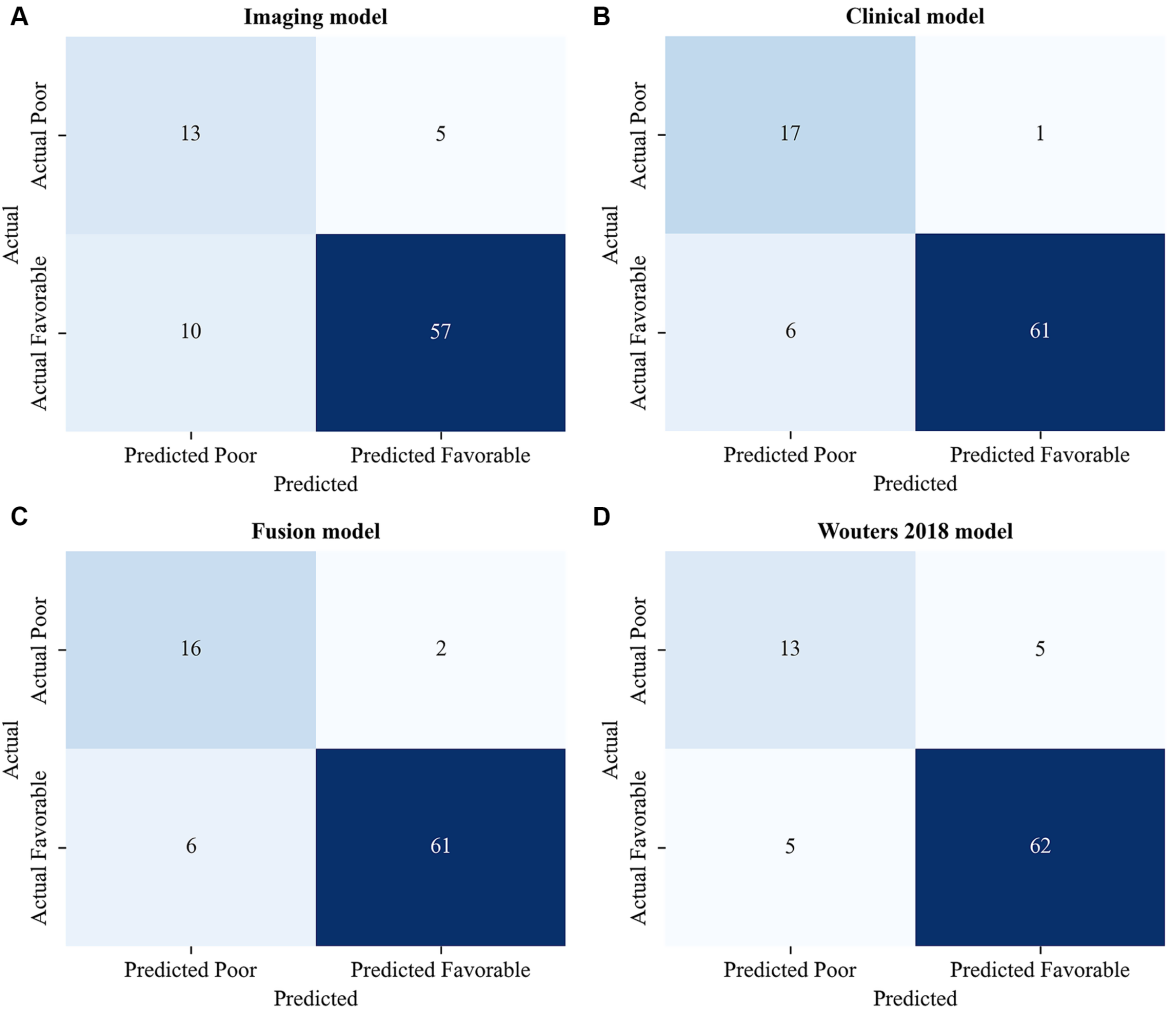
Confusion matrices of the four prediction models in the held-out internal test set. **(A)** Imaging model. **(B)** Clinical model. **(C)** Fusion model. **(D)** Wouters 2018 model.

Table 3 summarizes the performance of the Wouters 2018 model, clinical model, imaging model, and fusion model in the held-out internal test set. The fusion model had the highest numerical AUC among the four models (0.951, 95% CI: 0.908–0.994). However, DeLong testing showed that the AUC difference was statistically significant only between the fusion model and the imaging model (*p* = 0.038). The differences between the fusion model and the clinical model or the Wouters 2018 model were not statistically significant. Therefore, the incremental discriminative value of the fusion model over clinical modeling should be interpreted cautiously. At the threshold of 0.5, the clinical model showed the highest sensitivity, whereas the fusion model showed sensitivity between those of the clinical and imaging models. Confusion matrices for the four models are shown in Figure 6.

**Table 3:** Predictive performance of four models in the held-out internal test set.

Model	AUC (95% CI)	Sensitivity (95% CI)	Specificity (95% CI)
Wouters 2018 model	0.946 (0.890–0.972)	0.722 (0.493–0.875)	0.925 (0.835–0.967)
Clinical model	0.943 (0.888–0.985)	0.944 (0.742–0.990)	0.910 (0.819–0.957)
Imaging model	0.857 (0.764–0.949)	0.722 (0.493–0.875)	0.851 (0.747–0.917)
Fusion model	0.951 (0.908–0.994)	0.889 (0.672–0.969)	0.911 (0.819–0.957)

#### 3.2.4 Calibration analysis

Calibration analysis was performed to assess the agreement between predicted probabilities and observed outcome frequencies in the held-out internal test set. Among the evaluated models, the Wouters 2018 model showed the lowest Brier score, indicating the best overall calibration performance (Brier = 0.077), followed by the clinical model (Brier = 0.089), fusion model (Brier = 0.094), and imaging model (Brier = 0.122). The fusion model demonstrated better calibration than the imaging model but did not outperform the clinical model or the Wouters 2018 model based on the Brier score. In the calibration plot, the fusion model showed imperfect calibration, with apparent overestimation in the lower-risk range and underestimation in the higher-risk range. These findings suggest that the fusion model achieved moderate internal calibration but would require further calibration assessment, potential recalibration, and external validation. The calibration plots are shown in Figure 4.

#### 3.2.5 Decision curve analysis

Decision curve analysis was performed to evaluate the potential clinical utility of the four models in the held-out internal test set. The fusion model showed positive net benefit across threshold probabilities from 0.10 to 0.60 and remained above the treat-all and treat-none strategies throughout this range. The fusion model also showed consistently higher net benefit than the imaging model across the evaluated thresholds. However, the clinical model and the Wouters 2018 model showed comparable or higher net benefit than the fusion model across multiple threshold probabilities, and the Wouters 2018 model generally demonstrated the highest net benefit over much of the threshold range. These findings suggest that the fusion model may provide potential clinical value for internal risk stratification compared with imaging alone, but they do not demonstrate clear incremental clinical utility over the clinical model or the Wouters 2018 model. The decision curve analysis plots, including the enlarged view of the clinically relevant net-benefit range, are shown in Figure 5.

#### 3.2.6 Five-fold cross-validation

Stratified five-fold cross-validation was performed as a supplementary internal robustness analysis. The results are summarized in Table 4. The fusion model achieved a mean AUC of 0.940 ± 0.031, with sensitivity of 0.886 ± 0.067, specificity of 0.905 ± 0.038, and Brier score of 0.096 ± 0.021. These findings were directionally consistent with the held-out internal test set results, showing higher discrimination than the imaging model but no clear calibration advantage over the clinical model. However, this analysis remains an internal validation procedure and does not replace external validation.

**Table 4:** Stratified five-fold cross-validation performance.

Model	AUC, mean ± SD	Sensitivity, mean ± SD	Specificity, mean ± SD	Brier score, mean ± SD
Clinical model	0.928 ± 0.035	0.918 ± 0.061	0.887 ± 0.042	0.090 ± 0.019
Imaging model	0.846 ± 0.058	0.739 ± 0.094	0.846 ± 0.055	0.132 ± 0.028
Fusion model	0.940 ± 0.031	0.886 ± 0.067	0.905 ± 0.038	0.096 ± 0.021

### 3.3 Subgroup analysis of the fusion model

**Figure 7:**
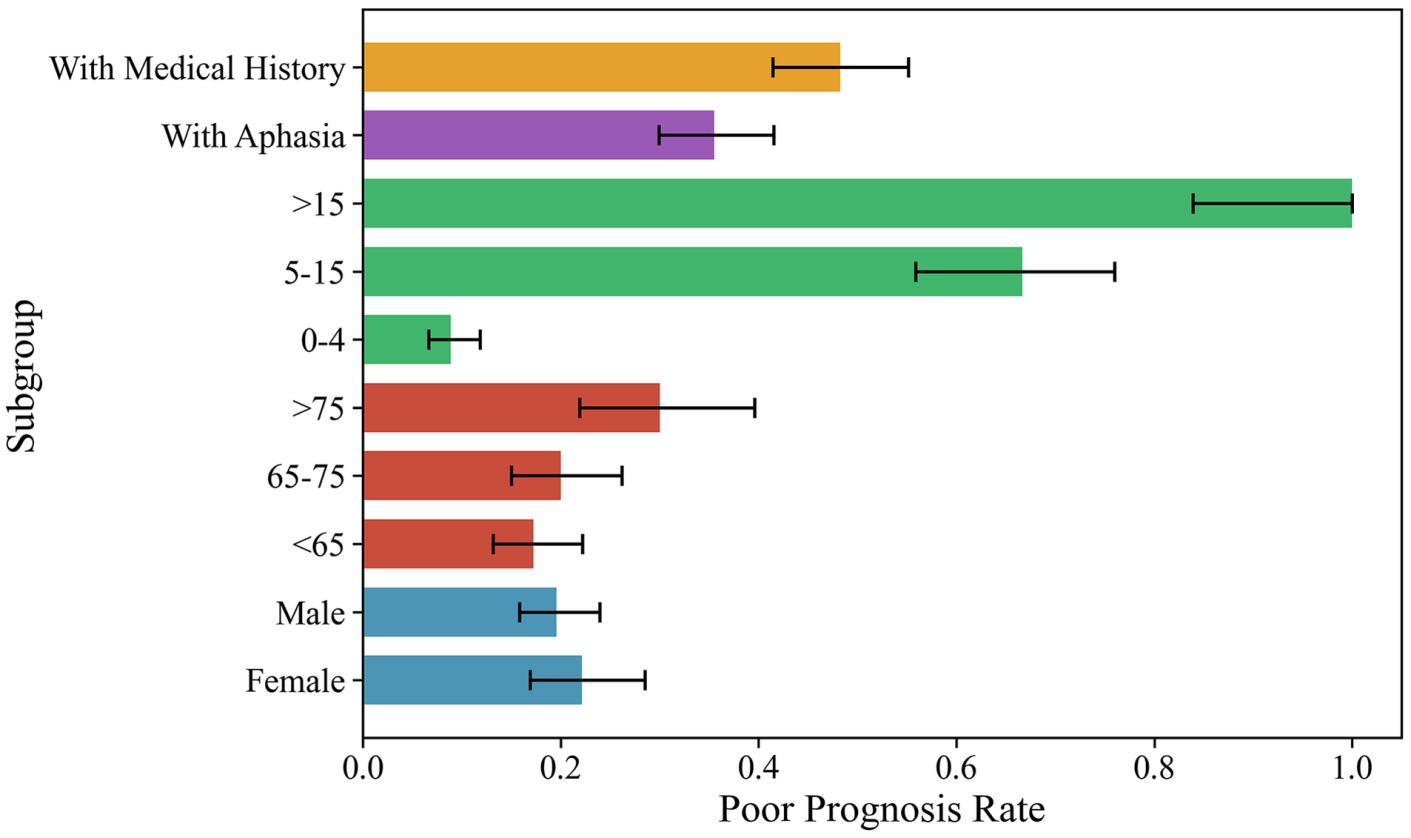
Subgroup analysis of the fusion model.

Subgroup analysis was conducted based on clinically relevant variables to evaluate clinical concordance of predicted risk patterns and to explore potential subgroup-specific variation in model performance (Figure 7). Table 5 shows the clinical concordance between observed poor outcome incidence and fusion model predicted risk across subgroups. Subgroup-based clinical concordance analysis showed clinically plausible risk patterns. The observed incidence of poor outcome and the mean predicted probability increased with age and baseline NIHSS score. Patients with medical history or aphasia had higher observed and predicted risks, whereas sex-related differences were relatively small. These findings support that the fusion model generated risk patterns broadly consistent with established clinical observations.

**Table 5:** Clinical concordance between observed poor outcome incidence and fusion model predicted risk across subgroups.

Subgroup variable	Category	*n*	Poor outcome, *n* (%)	Mean predicted probability
Age group	<65 years	267	46 (17.2)	0.176
Age group	65 to 75 years	195	39 (20.0)	0.203
Age group	>75 years	100	30 (30.0)	0.292
Baseline NIHSS score	0 to 4	461	41 (8.9)	0.094
Baseline NIHSS score	5 to 15	81	54 (66.7)	0.642
Baseline NIHSS score	>15	20	20 (100.0)	0.887
Medical history	No	361	18 (5.0)	0.092
Medical history	Yes	201	97 (48.3)	0.378
Aphasia	No	303	23 (7.6)	0.089
Aphasia	Yes	259	92 (35.5)	0.365
Sex	Male	368	72 (19.6)	0.204
Sex	Female	194	43 (22.2)	0.225

Formal interaction testing did not identify statistically significant interactions between model predicted risk and the predefined subgroup variables, including sex, age group, baseline NIHSS severity, medical history, and aphasia (Table 6). These results suggest no strong evidence of subgroup-specific performance heterogeneity in the present internal cohort. Because some subgroups contained limited numbers of poor outcome events, these subgroup analyses should be interpreted as exploratory.

**Table 6:** Formal interaction testing for subgroup-specific performance heterogeneity of the fusion model.

Subgroup variable	Categories used in interaction test	df	LR χ^2^	*p* for interaction
Sex	Male vs. female	1	0.14	0.708
Age group	<65, 65 to 75, >75 years	2	1.55	0.461
Baseline NIHSS severity	0 to 4 vs. >4	1	1.17	0.279
Medical history	No vs. yes	1	0.70	0.403
Aphasia	No vs. yes	1	0.43	0.512

### 3.4 Model interpretability and feature visualization

**Figure 8:**
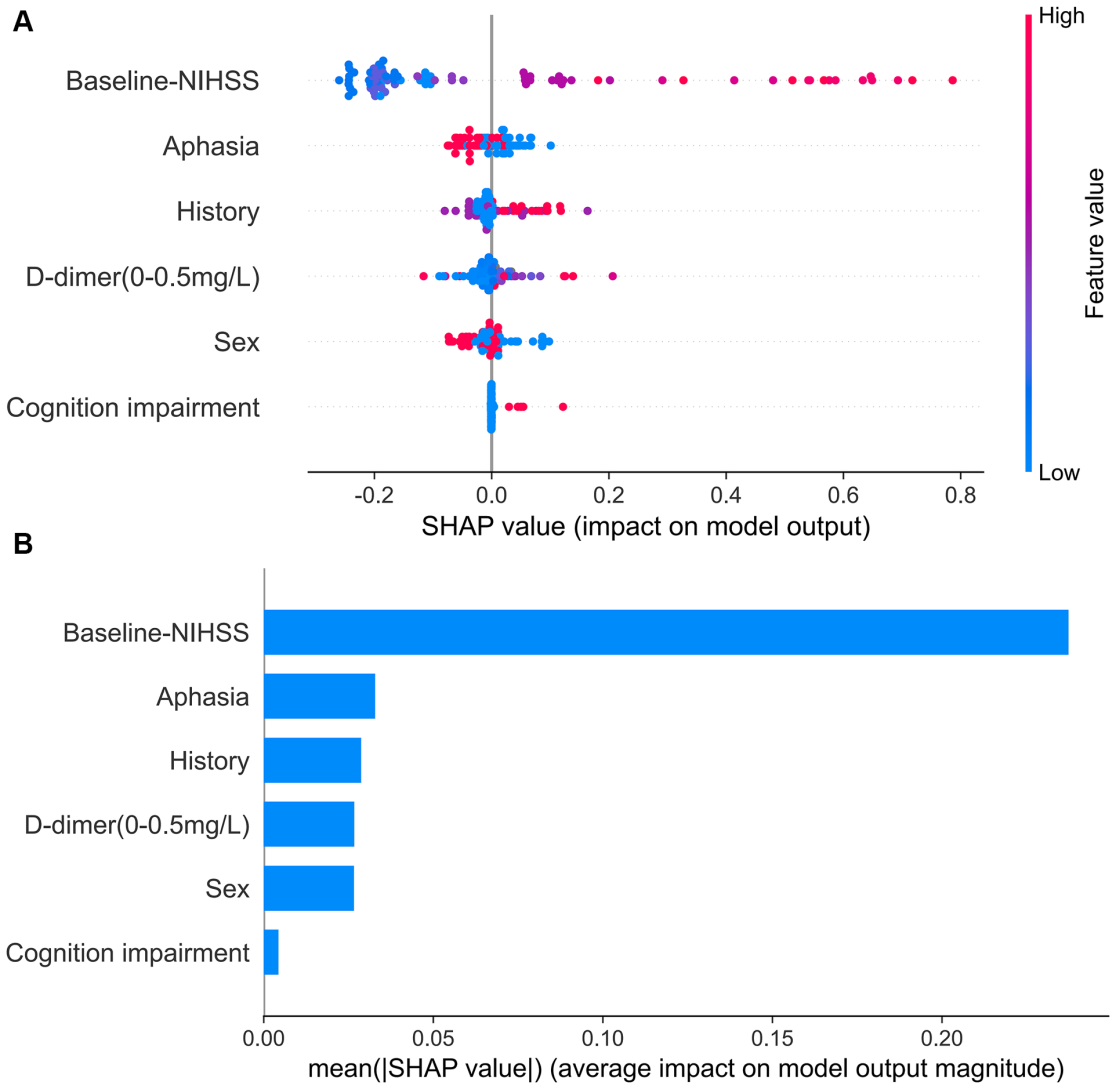
SHAP analysis of the clinical model for predicting poor functional outcome in AIS patients. **(A)** SHAP summary plot. **(B)** SHAP feature importance bar plot.

The SHAP analysis of the clinical model provided an estimate of the relative contribution and direction of each clinical variable in predicting poor functional outcome (Figure 8). In the SHAP summary plot, each point represents an individual patient, with red indicating higher feature values and blue indicating lower values (Figure 8A). Baseline NIHSS scores showed the greatest influence on the model output, with higher values corresponding to positive SHAP values and a higher predicted risk of poor prognosis. Aphasia, previous medical history, and D-dimer levels also contributed positively to the prediction, whereas sex and cognitive impairment showed relatively smaller and less consistent effects. The SHAP feature importance bar plot (Figure 8B) further confirmed that the baseline NIHSS score was the most influential predictor, followed by aphasia, previous medical history, and D-dimer levels, indicating that the baseline severity of neurological deficits plays a key role in stroke prognosis prediction.

**Figure 9:**
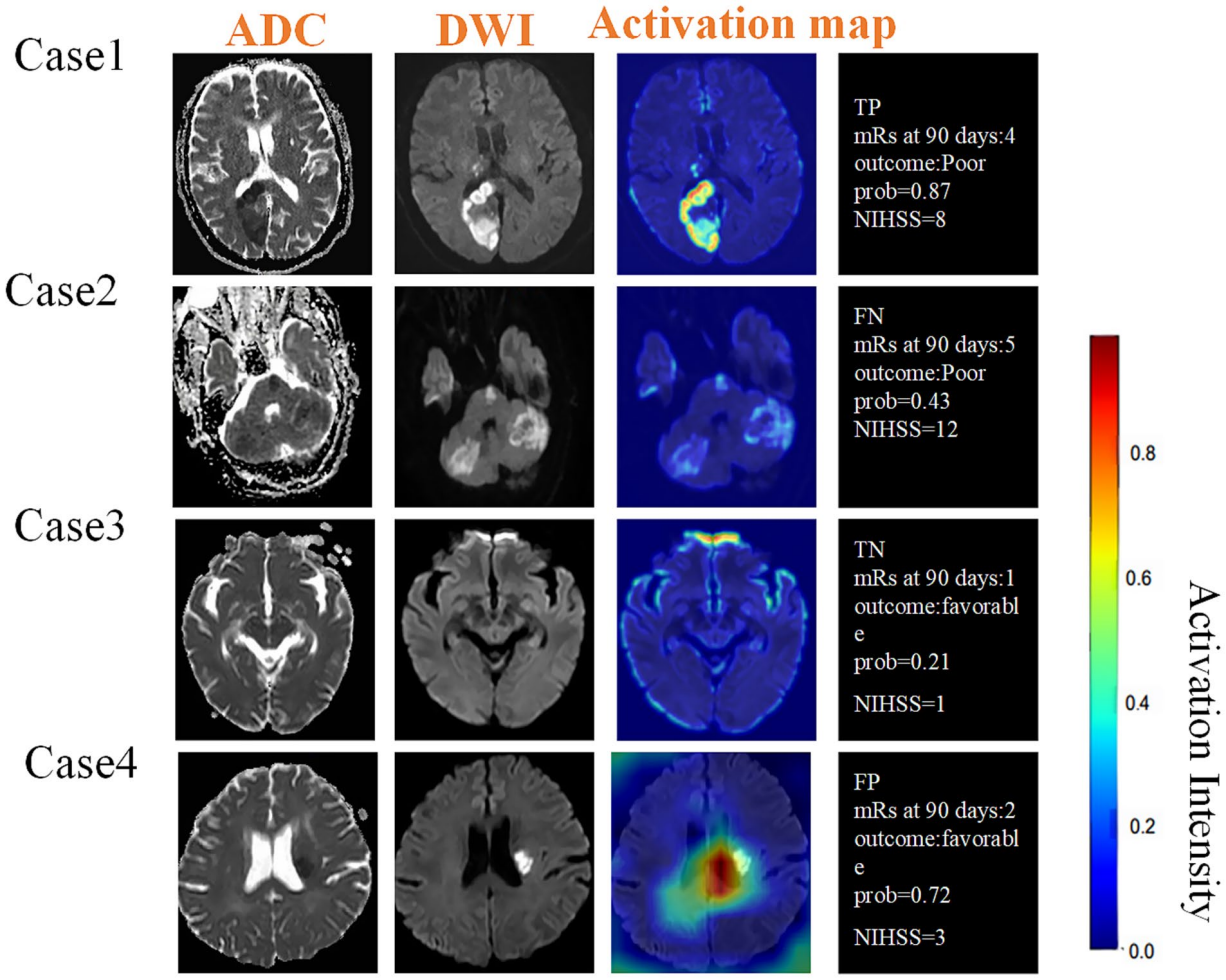
Visualization of the mean activation heatmap of the imaging model.

To qualitatively assess the imaging model’s response patterns, channel-wise averaged activation heatmaps were generated for multiple representative cases from the held-out internal test set, including true-positive (TP, *n* = 1), false-positive (FP, *n* = 1), true-negative (TN, *n* = 1), and false-negative (FN, *n* = 1) predictions (Figure 9). In the TP case (correctly predicted poor outcome), high-activation regions overlapped well with the hyperintense DWI lesions and corresponding ADC hypointense areas. The FP case (predicted poor but actual favorable outcome) showed moderate activation that partly overlapped with non-infarct regions. In the TN case (correctly predicted favorable outcome), activation was generally low and scattered without clear lesion-like patterns. For the FN case (missed poor outcome), activation was weaker and less focused on the actual infarct area compared with the TP case. These observations are qualitative and exploratory; they do not serve as a definitive validation of lesion localization.

## 4 Discussion

This retrospective single-center study developed and internally evaluated a probability-level stacked ensemble model integrating DWI and ADC MRI-based imaging predictions with routinely available clinical predictions to predict 90-day poor functional outcome in patients with AIS. The fusion model achieved the highest numerical AUC in the held-out internal test set and significantly outperformed the imaging model, but it did not show a statistically significant AUC improvement over the clinical model or the Wouters 2018 model. The clinical model also showed the highest sensitivity at the prespecified threshold. In addition, calibration and decision curve analyses did not demonstrate a consistent incremental advantage of the fusion model over the clinical model or the Wouters 2018 model (27). One plausible explanation is that the baseline NIHSS score already captures the overall severity of early neurological deficits, thereby accounting for a large proportion of the prognostic signal that may also be partly reflected by acute DWI-ADC abnormalities. In this moderate-sized single-center cohort with a limited number of poor-outcome events in the held-out internal test set, this overlap may have limited the measurable incremental contribution of the imaging-derived probability, particularly for calibration and net-benefit estimates. Therefore, the main finding should be interpreted as high internal discrimination and improved performance compared with imaging alone, rather than definitive superiority over clinical prediction models.

The present findings are consistent with recent studies showing that multimodal imaging and clinical modeling can improve AIS outcome prediction, particularly when compared with imaging-only approaches (28). However, our results also support a more cautious interpretation, because strong clinical predictors can already capture substantial prognostic information (19). Previous studies have shown that age, NIHSS score, stroke history, D-dimer, and other clinical factors are associated with 90-day functional outcomes, and some studies have reported comparable performance between machine learning and regression-based models (10, 29). In our study, SHAP analysis also identified baseline NIHSS score as the dominant clinical contributor, followed by aphasia, medical history, and D-dimer level. These findings are clinically plausible, but SHAP values should be interpreted as model attribution rather than causal effects (16, 30). Compared with recent multimodal AIS prognostic studies, the present model achieved a numerically high internal test AUC of 0.951, which is close to the AUC reported by Jung et al. (13) for multimodal ensemble deep learning. However, direct numerical comparisons should be made cautiously because study populations, imaging modalities, outcome definitions, validation designs, and treatment contexts differed across studies. Pei et al. focused on prognosis prediction after mechanical thrombectomy using multimodal MRI radiomics and deep learning (14), whereas the present study evaluated DWI-ADC MRI and routinely available clinical variables in a broader single-center AIS cohort. In contrast to more complex attention-based multimodal architectures, such as the cross-attention approach reported by Amador et al. (18), our probability-level stacking strategy was intentionally simpler and was selected to reduce model complexity and improve transparency in a moderate-sized single-center dataset. Therefore, the contribution of the present study is not the proposal of a fundamentally new fusion algorithm, but the internal evaluation of a transparent probability-level fusion framework for DWI-ADC MRI and clinical risk modeling in AIS.

These calibration and decision curve findings further indicate that discrimination alone is insufficient to support clinical applicability (31). Although the fusion model improved on the imaging model, its calibration and net-benefit results did not show a consistent advantage over the clinical model or the Wouters 2018 model. This suggests that the added imaging branch improved risk discrimination relative to imaging alone but did not translate into better probability reliability or clinical utility than strong clinical risk modeling in this cohort. Therefore, recalibration and external validation are needed before broader application. The subgroup analysis showed clinically plausible risk patterns and no statistically significant interaction between model-predicted risk and predefined subgroup variables, but these analyses were exploratory. In the present study, activation heatmaps were generated for representative TP, FP, TN, and FN cases to qualitatively assess model response patterns across different prediction outcomes (32).

Several limitations should be acknowledged. First, this was a retrospective single-center study without external multicenter validation. Although a held-out internal test set and five-fold cross-validation were used, these procedures cannot replace independent external validation. Second, potential overfitting remains an important concern because model development, hyperparameter selection, probability-level fusion, and evaluation were all performed within the same institutional cohort, and the held-out internal test set contained only 18 poor outcome events. These factors may reduce the precision of sensitivity, specificity, calibration, decision curve analysis, and subgroup interaction estimates. Third, treatment-related variables, stroke subtype, recanalization status, infarct volume, rehabilitation intensity, and systematic lesion annotations were not fully incorporated. Fourth, the current heatmap analysis was qualitative and based on representative cases only. Therefore, the fusion framework should be viewed as an internally evaluated risk stratification approach whose incremental clinical value requires confirmation through external validation, recalibration, and prospective evaluation.

## 5 Conclusion

Overall, probability-level fusion of DWI-ADC MRI-based imaging predictions and routinely available clinical predictions provided an internally evaluated framework for 90-day poor functional outcome risk stratification in patients with AIS. The fusion model showed high internal discrimination and improved performance compared with the imaging model alone. However, it did not demonstrate a statistically significant AUC improvement, better calibration, or a consistent decision curve advantage over the clinical model or the Wouters 2018 model. These findings suggest that the fusion framework may be useful for internal risk stratification, but its incremental clinical value requires confirmation through external multicenter validation, recalibration, and prospective evaluation.

## Data Availability

The original contributions presented in the study are included in the article/supplementary material, further inquiries can be directed to the corresponding authors.
